# Academic Stress, Physical Activity, Sleep, and Mental Health among Chinese Adolescents

**DOI:** 10.3390/ijerph18147257

**Published:** 2021-07-07

**Authors:** Xihe Zhu, Justin A. Haegele, Huarong Liu, Fangliang Yu

**Affiliations:** 1 Department of Human Movement Sciences, Old Dominion University, Norfolk, VA 23529, USA; x2zhu@odu.edu (X.Z.); jhaegele@odu.edu (J.A.H.); 2 School of Physical Education, China University of Geosciences, Wuhan 430074, China; liuhuarong@cug.edu.cn; 3 School of Sport Training, Nanjing Sport Institute, Nanjing 210014, China

**Keywords:** academic burden, anxiety, depression, mediation, youth, prevalence

## Abstract

The purpose of this study was to examine the impacts of academic stress on physical activity and sleep, and subsequently their impacts on anxiety and depression. Methods: This cross-sectional study collected data from a convenience sample of 1533 adolescents in an eastern province in China. Surveys were used to collect data on academic stress, anxiety, depression, sleep, physical activity, and demographics. Descriptive statistics, correlation analysis, and path analysis were used to analyze data. Results: The participants reported about 6.77 ± 0.89 h of sleep per day and 1.62 ± 1.79 days of 60 min of physical activity each week. Academic stress was positively correlated with anxiety and depression, which were negatively correlated with physical activity and sleep. The path analysis showed that academic stress directly predicted anxiety (β = 0.54) and depression (β = 0.55), and hours of sleep (β = 0.024) and the number of days of 60 min physical activity (β = 0.014) mediated the relation. Conclusion: The results largely supported our hypotheses and supported the need to lessen academic stress experienced by Chinese adolescents, in effort to enhance mental health indices directly, and by allowing for engagement in health-related behaviors such as physical activity and sleep.

## 1. Introduction

Adolescence is a critical developmental period for mental health disorders, with a high percentage of all lifetime mental health disorders being identified for the first time by 14 years of age [1]. Anxiety, characterized as excessive fear or worry, and depression, characterized by persistent sad or irritable mood, are of particular concern since they have been identified as being the most diagnosed mental health challenges during adolescence [2,3]. Highlighting this, a meta-analysis of 51 studies involving 144,060 secondary school students in mainland China indicated an estimated incidence of depression of 24.3% [3]. Similarly, in a survey study of 1576 secondary students in China, Hesketh and Ding [4] found that a considerable portion of the student body displayed symptoms of anxiety severe enough to interfere with basic life functions (i.e., sleep) as well as quality of life. These figures are alarming, given that anxiety and depression during childhood has been associated with a litany of deleterious outcomes, such as drug and alcohol abuse, suicide risk, and other physical health problems [5]. Of additional concern, the identification of mental health disorders such as anxiety and depression during adolescence is strongly associated with increased risk of experiencing mental health concerns across the lifespan [6].

Given the high prevalence, early onset, and detrimental effects of mental health disorders such as anxiety and depression, mental health disorders among adolescents are considered a major public health concern in China and worldwide [7,8]. Gaining an understanding of factors that can help reduce the likelihood of experiencing mental health disorders such as anxiety and depression among adolescents is of scholarly and clinical significance. Physical activity and sleep duration are two volitational, lifestyle-related behaviors that have been identified as being associated with the prevalence of anxiety and depression among adolescents [9,10,11,12,13]. For example, in an analysis of data from 20,708 adolescents aged 12–17 in the U.S. from the 2016 National Survey of Children’s Health (NSCH), Zhu et al. [13] found that those who engaged in zero days per week of physical activity were over twice as likely to have anxiety and depression as peers engaging in daily physical activity for at least 60 min per day. Similarly, data analyzed from the National Comorbidity Survey Adolescent Supplement, a nationally represented cross-sectional survey of 10,123 adolescents in the U.S., showed that suboptimal sleep patterns were significantly associated with a variety of deleterious psychological outcomes, including anxiety, behavioral disorders, and poor perceived mental health [12]. Despite these explicated psychological benefits [12,13,14], youth may not be engaging in recommended durations of physical activity or sleep. Of concern, a recent examination of health-related behaviors among 1338 adolescents aged 14–19 in an eastern China province demonstrated that just 2.8% and 7.9% met age-appropriate physical activity and sleep duration guidelines, respectively [15]. Since youth are not engaging in adequate amounts of physical activity or sleep, it is likely that they are not receiving the full psychological benefits associated with these health-related behaviors.

To ensure that youth are receiving the psychological benefits associated with adequate amounts of physical activity and sleep duration, it is critical to identify factors that contribute to these low proportions of recommendation adherences. Academic stress, which is associated with the notably high learning/testing standards, outcomes, and expectations of secondary schools in China, may be one factor that may be of interest in examining health-related behaviors and mental health among Chinese adolescents. Academic stress has been identified as the most stressful aspect of education that most students who attend secondary schools in China experience, with stress peaking during the final years of junior and senior secondary schools [16]. This type of academic stress, and associated academic burden/pressure, may play a significant role in why youth in China engage in inadequate amounts of health-related behaviors [17], and it may also influence high incidences of anxiety and depression among students directly [18]. Supporting this assertion, Greenberger et al. (2000) noted that the association between academic stress and poor mental health appears to be stronger among Chinese students when compared to those in other countries [19]. 

In summary, the explicated empirical findings suggest that higher levels of academic stress may be directly associated with higher levels of anxiety and depression [18], and that it may be indirectly related to them through its negative impact on physical activity and sleep [17], which in turn are associated with anxiety and depression among adolescents [12,13,14]. In this sense, physical activity and sleep may act as mediators for the relationship between academic stress and anxiety and depression. To examine these relationships, the purpose of this study was to examine the impacts of academic stress on physical activity and sleep, and subsequently their impacts on anxiety and depression. We hypothesized that higher levels of academic stress would be directly associated with higher levels of anxiety and depression. In addition, we hypothesized that that higher levels of academic stress would be associated with lower levels of weekday physical activity and sleep duration, which in turn would be associated with higher levels of anxiety and depression.

## 2. Methods

### 2.1. Study Design and Sample

This cross-sectional study collected data from a convenience sample of adolescents in a large regional high school in an eastern province in China. This high school had an enrollment of approximately 4500 high school-aged students, 1533 of which consented and participated in this data collection (35.9% response rate). Data were collected via self-reported survey during the fall semester (November–December) of 2019. The corresponding author’s institutional review board (human subjects research ethics committee) and the local school reviewed and approved the study protocols. Parental consent was sought for all potential participants under 18 years of age, and assent was obtained from all participants prior to data collection. 

### 2.2. Measures

#### 2.2.1. Academic Stress

The 16-item Educational Stress Scale for Adolescents (ESSA) was used to measure participants’ academic stress. ESSA was developed through a series of cross-sectional surveys with more than 2000 Chinese adolescents measuring the factors related to academic stress such as pressure to study, school workload, worry about grades, grade expectation, and despondency [20]. ESSA is a five-point Likert type scale with responses ranging from 1 = “strongly disagree” to 5 = “strongly agree”, with a higher score indicating a higher level of stress. Example items from ESSA include “I feel a lot of pressure in daily study,” and “I feel there is too much homework.” Research shows that ESSA has a good factorial tenability and good internal consistency (Cronbach α = 0.81) and test–retest reliability (ρ = 0.78) [20]. In this study, we used the grand composite of ESSA to measure academic stress as a continuous variable.

#### 2.2.2. Anxiety and Depression

We measured the level of anxiety using the Chinese version of General Anxiety Disorder-7 (GAD-7). GAD-7 is a four-point Likert-type scale containing 7 items with responses from 0 = “not at all” to 3 = “nearly every day”. The leading question for GAD-7 asks, “how often have you been bothered by the following over the past 2 weeks?” An example item from GAD-7 reads “not being able to stop or control worrying?” The total scores of 5, 10, and 15 are recommended as cut-offs for mild, moderate, and severe anxiety, respectively [21]. GAD-7 has been reported with good concurrent validity (72% sensitivity for social anxiety disorder), and test–retest reliability (ρ = 0.83) [21]. The level of depression was measured using the Patient Health Questionnaire (PHQ-9), which is a four-point Likert-type scale containing 9 items [22]. The leading question for PHQ-9 asks, “how often have they been bothered by the following over the past 2 weeks?” An example item reads “little interest or pleasure in doing things?” Four response options are available with 0 = “not at all” to 3 = “nearly every day”. Total scores of 9, 14, and 20 are cut-offs for mild, moderate-to-severe, and severe levels of depression. PHQ-9 has shown to have good concurrent validity (88% sensitivity for major depression) [22]. The cut-offs were used for frequency analyses for anxiety and depression, and GAD-7 and PHQ-9 scores were used as continuous variables for the path analysis. 

#### 2.2.3. Demographics

Demographic data were collected via a four-item survey which included questions pertaining to the participants’ age, ethnicity, sex, and parents’ highest education. Age was collected as chronological age, sex asked participants to identify as male or female, and ethnicity included options of Han or minority populations. Participants selected from three options to identify their parent’s education, including (a) high school or less, (b) some college or bachelor’s degree, and (c) graduate or professional degree. 

#### 2.2.4. Physical Activity and Sleep

Physical activity and sleep were each measured using one-item that was adopted from the National Survey of Children’s Health from the U.S. Census Bureau (2019). For physical activity, adolescents were asked: “During the past week, on how many days did you exercise, play a sport, or participate in PA (that resulted in elevated heart rate, accelerated breathing, and/or sweating) for at least 60 min?”. Eight response options were available, ranging from “0” to “7” days. For sleep, participants were asked an open-ended question, “during the past week, how many hours of sleep did you have on an average weeknight?” Nine response options were available, ranging from “4” to “12” hours. The selected numerical values were used to indicate the number of physical active days (for 60 min or more) and the number of sleeping hours. 

### 2.3. Data Analysis

**Figure 1:**
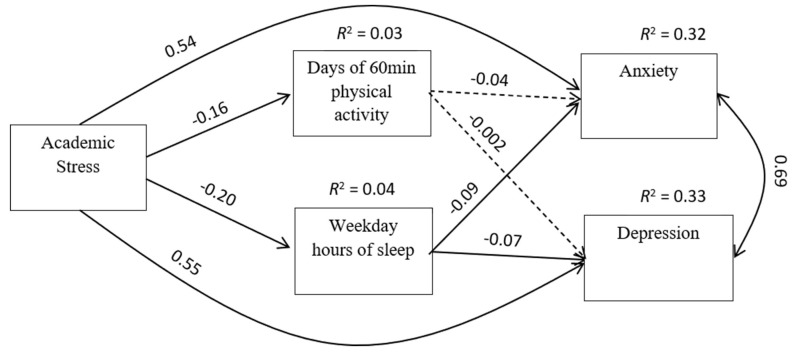
Relations between academic stress, physical activity, sleep, and mental health (dotted lines are not statistically significant, *p* > 0.05).

We analyzed the data in three steps. In the first step, we ran descriptive statistical analyses such as frequency analysis, variable central tendency, and variability. Then, we screened the ratio variables for statistical assumptions such as variance homogeneity and multivariate kurtosis and ran a Pearson product–moment correlation analysis. Finally, to test our hypotheses (as shown in Figure 1), we ran a path analysis based on variance–covariance structure. Multiple goodness of fit indices were used during the model testing processes, which included the χ^2^, normed comparative fit index (CFI; >0.95 great, >0.90 traditionally acceptable), the root mean square error of approximation (RMSEA; <0.05 great, 0.05–0.10 is acceptable, >0.10 poor), and standardized root mean square residual (SRMR; <0.09 is acceptable). These indices reflect model-data fit (χ^2^), absolute fit (SRMR, RMSEA), incremental fit (CFI), and the corresponding thresholds are generally accepted in path analysis/structural equation modeling [23,24,25]. Additionally, Wald’s z and Lagrange Multiplier (LM) tests were used to elicit possible model re-specifications. The extent to which each newly modified model was an improvement over its predecessor was assessed by Δχ^2^ and ΔCFI between two models, whereby (Δχ^2^) *p* < 0.05 and Δ|CFI| > 0.01 is considered significant [26]. The data analyses were conducted using EQS 6.3 [27].

## 3. Results

As shown in Table 1, among the 1533 participants aged between 14 and 19 years old, 15.8% reported moderate to severe levels of general anxiety, and 7.0% reported moderately severe to severe depression. Based on GAD-7 and PHQ-9 data, a vast majority of participants reported minimal to mild anxiety (84.2%) and mild to moderate depression (93.0%). Consistent with the general population, most participants were Han ethnic (97.6%), and about half were female (49.2%). About 7.2% of participants’ parents had graduate or professional degrees, 47.1% had some college or a bachelor’s degree, and 45.7% had high school or less education. 

The participants reported relatively low levels of physical activity and hours of sleep. On average, as shown in Table 2, the participants reported about 6.77 ± 0.89 h of sleep per day and 1.62 ± 1.79 days of having the suggested 60 min of daily physical activity each week. The measures of academic stress (ESSA), anxiety (GAD-7), and depression (PHQ-9) showed great internal consistency with Cronbach α values being 0.92, 0.93, and 0.89, respectively. The skewness and kurtosis of the variables are approximately normal. Academic stress was positively correlated with anxiety (r = 0.57) and depression (r = 0.56). As shown in Table 2, average hours of sleep were negatively correlated with anxiety (r = −0.20) and depression (r = −0.18), and the number of days of 60 min physical activity were negatively correlated with anxiety (r = −0.14) and depression (r = −0.10) as well. 

The path analysis showed that the data fit the hypothesized model well, χ^2^ = 995.85, df = 48, CFI = 0.996, NNFI = 0.961, SRMR = 0.019, RMSEA = 0.076 (90% CI: 0.041–0.119). As displayed in Figure 1, hours of sleep and the number of days of 60 min physical activity were negatively associated with anxiety and depression, although the direct path from physical activity to anxiety or depression was not statistically significant (*p* > 0.05, the dotted lines in Figure 1). Academic stress directly predicted anxiety (β = 0.54), depression (β = 0.55), and hours of sleep (β = 0.024), and the number of days of 60 min physical activity (β = 0.014) mediated the relation (Table 3, indirect paths). Overall, the model explained about 32% of the variances in anxiety and 33% of that in depression. 

## 4. Discussion

Academic stress plays a prominent role in the lives of adolescents who attend secondary schools in China [16,17] and may have deleterious direct and indirect associations with anxiety and depression [18]. As such, the purpose of this study was to examine the impacts of academic stress on physical activity and sleep and subsequently their impacts on anxiety and depression. A number of interesting results emerged from this study. For example, similar to previous research [12,13,14,15], participants reported engaging in suboptimal hours of sleep and few days of 60 min of physical activity per week, on average. Interestingly, though, the prevalence of reporting moderate to severe anxiety (15.8%) and moderately severe to severe depression (7.0%) were lower than expected based on previous literature [3,4,5]. For example, among 1576 respondents in Hesketh and Ding’s [4] analysis in two regions in China, 70% claimed to worry a lot, with nearly half noting that this interfered with their enjoyment of life. Discrepancies in prevalence rates may be a result of a number of different factors, including differences in the measurement and conceptualization of the variables, as well as the collection of data in different regions of China. Regardless, though, the prevalence of moderate to severe anxiety and moderately severe to severe depression in this study, as well as deleterious outcomes associated with anxiety and depression during childhood [5] were still considerable enough to warrant exploration into associations with physical activity, sleep duration, and academic stress. 

Overall, the data showed good fit to the hypothesized model, which was largely supported our hypotheses. Specifically, our results showed that academic stress was directly, significantly positively associated with both anxiety (β = 0.54) and depression (β = 0.55) among this sample, supporting the first hypothesis that higher academic stress is associated with higher anxiety and depression levels. This direct path has logical and empirical support, where previous research has identified an association between academic stress and poor mental health among Chinese students [19]. In addition, our results generally support the hypothesized indirect path between academic stress, physical activity and sleep, and anxiety and depression. Specifically, our results showed that academic stress was negatively related to the number of days of 60 min of physical activity (β = −0.16) and weekday hours of sleep (β = −0.20). This result provides data that suggests that in addition to directly influencing anxiety and depression [19], academic stress, and associated constructs such as academic burden and academic pressure, can play a significant role in reducing the likelihood of adolescents in China engaging in adequate health-related behaviors such as physical activity and sleep [17]. The implications for Chinese youth are clear, where academic stress is associated with high learning/testing standards, and expectations may be reducing the time available for or students’ ability to engage in health-related behaviors [15] as well as inhibiting mental health in general [19]. This problem may be particularly evident during transition grades (e.g., 6th, 9th, and 12th grade), where academic burden and stress peaks [16], and therefore, adolescents have cited having little time to engage in health-related behaviors [17]. Based on this, it is unsurprising that few participants in this study, and in previous research [15], have been shown to meet the recommended age-appropriate physical activity and sleep duration guidelines. Reduced engagement in health-related behaviors can have additive effects, influencing undesirable outcomes such as higher rates of overweight or obesity [28,29]. As such, academic stress may pose a significant problem among Chinese adolescents, given the deleterious effects it may have on anxiety, depression, and health-related behaviors. Therefore, intervention research exploring avenues to reduce academic stress in this population is warranted. Given their negative associations with academic stress, physical activity and sleep could be viable behavioral options for such interventions based on previous findings [17]. 

Considering relationships between academic stress and physical activity and sleep duration, we further hypothesized that low levels of these health-related behaviors would be associated with higher levels of anxiety and depression. As shown in Figure 1, hours of sleep were significantly negatively associated with anxiety (β = −0.09) and depression (β = −0.07), supporting our hypothesis. However, days of 60 min of physical activity was not significantly associated with either anxiety (β = −0.04) or depression (β = −0.002). This finding was unexpected, given the central and prominent role that physical activity generally plays as a health-related behavior that influences mental health, as well as physiological outcomes, among youth [9,10,11,12,13,14,28,29,30]. We can speculate here, though, that perhaps the average physical activity levels of the participants may not have been sufficient to elicit improvements in anxiety and depression scores among our participants, reducing the likelihood of significant results. Additionally, the limitations of the physical activity measure (discussed below), which was not a direct measure of time, could be the culprit for the insignificant relations as it lacks the levels of measurement discrimination. Future research interested in understanding the impact that health behaviors, such as physical activity and sleep, can play on anxiety and depression may elect to consider an integrative approach, such as the 24-movement framework [15,28], to explore the integrative or additive effects of engaging in sufficient levels of more than one movement behavior. Future studies may also expand to other health-related behaviors, such as nutrition, drug usage, or screen time, to gain a more comprehensive understanding of the associations between various health-related behaviors and mental health among adolescents [15,28,29].

From a clinical/practical perspective, the findings point to an important element for the service providers of this population. That is, clinicians may explore and look at the adolescents’ academic stress, and potentially their behaviors such as physical activity and sleep, as these variables could be useful supplemental services for those suffering from anxiety or depression disorders. 

The current study benefited from several strengths (e.g., the use of a relatively large sample size in an understudied geographic area). However, several imitations should be acknowledged. First, the convenience sample included in this analysis was from just one large high school in an eastern province in China, limiting the generalizability of the findings. Second, the utilization of self-report measures to measure physical activity and sleep may be viewed as a limitation. While self-report instruments have historically acted as a primary source of data for health-related variables such as physical activity and sleep, there are concerns that social desirability can influence respondents to overreport desirable behaviors [31]. The recall on number of days may also limit the level of measurement discrimination for physical activity and sleep, thus failing to detect a potential directional relationship. The utilization of a brief self-report was advantageous, as it shortened the survey length allowing for higher completion rates and allowed access to a large group of individuals in an economically efficient way that would not be otherwise feasible if using objective monitors. In addition, just one question for physical activity and sleep was used, making it challenging to evaluate the reliability of responses. Furthermore, there are potentially other confounding variables such as the adolescents screen viewing and study times, which may have an impact on the variables in the study but were not collected. Finally, because this study utilized a cross-sectional survey design, casual relationships among study variables may not be inferred. Longitudinal or intervention studies are needed to support the current findings. 

## 5. Conclusions

This study examined the impacts of academic stress directly on anxiety and depression, and indirectly through physical activity and sleep. Overall, the path analysis showed that the data fit our hypothesized model well, and the overall model explained 32% and 33% in the variance in anxiety and depression, respectively. As such, this study provides support for assertions about the adverse impacts that academic stress can have on anxiety and depression directly, and for physical activity and sleep as mediators of this relationship. The findings here warrant further work that explores mechanisms to reduce academic stress among adolescents in China, whom have been found to experience this stress at high rates in prior studies [16], in order to improve mental health indices. 

## Figures and Tables

**Table 1:** Demographic characteristics of adolescents in high school.

Age Range	14–19 Years Old N = 1533
Age (y), mean (SD)	16.67 (0.69)
Gender (%, 95% CI)	
Female	49.2% (46.6–51.8%)
Male	50.8% (48.2–53.4%)
Ethnicity (%, 95% CI)	
Han	97.6% (96.7–98.3%)
Minority	2.4% (1.7–3.3%)
Parent education (%, 95% CI)	
High school or less	45.7% (43.2–48.3%)
Some college or bachelor’s degree	47.1% (44.4–49.6%)
Graduate or professional degree	7.2% (5.9–8.6%)
Anxiety (%, 95% CI)	
Minimal to mild	84.2% (82.6–85.9%)
Moderate to severe (GAD-7 > 9)	15.8% (14.1–17.4%)
Depression (%, 95% CI)	
Mild to moderate	93.0% (91.7–94.2%)
Moderately severe to severe (PHQ-9 > 14)	7.0% (5.8–8.3%)

CI: confidence interval; SD: standard deviation.

**Table 2:** Mean and correlation of academic stress, physical activity, sleep, anxiety and depression.

Variable	Mean ± SD	Min	Max	α	Skewness	Kurtosis	1	2	3	4
1. Academic Stress (total point)	46.36 ± 12.75	16	80	0.92	−0.05	0.22	1			
2. 60 min of PA (# days/week)	1.62 ± 1.79	0	7		1.19	0.99	−0.16 **	1		
3. Sleep (# hour/day)	6.77 ± 0.89	2	10		−0.66	2.38	−0.20 **	0.11 **	1	
4. Anxiety (GAD-7)	5.11 ± 4.94	0	21	0.93	1.21	1.25	0.57 **	−0.14 **	−0.20 **	1
5. Depression (PHQ-9)	6.18 ± 5.22	0	27	0.89	1.29	2.17	0.56 **	−0.10 **	−0.18 **	0.79 **

Note: ** *p* < 0.01, PA = physical activity.

**Table 3:** Decomposition of standardized direct and indirect effects of academic stress.

Endogenous Variable	Direct	Indirect	Total
Physical activity	−0.16		−0.16
Sleep	−0.20		−0.20
Anxiety (GAD-7)	0.543	0.024	0.567
Depression (PHQ-9)	0.549	0.014	0.563

## Data Availability

The data presented in this study are available from the corresponding author. The data are not publicly available due to privacy and ethical considerations.
